# The Effect of Changing the Contraction Mode During Resistance Training on mTORC1 Signaling and Muscle Protein Synthesis

**DOI:** 10.3389/fphys.2019.00406

**Published:** 2019-04-18

**Authors:** Satoru Ato, Daisuke Tsushima, Yurie Isono, Takeshi Suginohara, Yuki Maruyama, Koichi Nakazato, Riki Ogasawara

**Affiliations:** ^1^Department of Life Science and Applied Chemistry, Nagoya Institute of Technology, Nagoya, Japan; ^2^Department of Life Sciences, The University of Tokyo, Tokyo, Japan; ^3^Department of Exercise Physiology, Nippon Sport Science University, Tokyo, Japan

**Keywords:** skeletal muscle, resistance exercise, eccentric contraction, cell signaling, mTOR, P70S6K, 4E-BP1

## Abstract

Acute resistance exercise (RE) increases muscle protein synthesis (MPS) via activation of mechanistic target of rapamycin complex (mTORC), and chronic resistance exercise training (RT) results in skeletal muscle hypertrophy. Although MPS in response to RE is blunted over time during RT, no effective restorative strategy has been identified. Since eccentric muscle contraction (EC) has the potential to strongly stimulate mTORC1 activation and MPS, changing the muscle contraction mode to EC might maintain the MPS response to RE during chronic RT. Male rats were randomly divided into RE (1 bout of RE) and RT (13 bouts of RE) groups. Additionally, each group was subdivided into isometric contraction (IC) and EC subgroups. The RE groups performed acute, unilateral RE using IC or EC. The RT groups performed 12 bouts of unilateral RE using IC. For bout 13, the RT-IC subgroup performed a further IC bout, while the RT-EC subgroup changed to EC. All muscle contractions were induced by percutaneous electrical stimulation. Muscle samples were obtained at 6 h post exercise in all groups. After the 1st RE bout, the EC group showed significantly higher p70S6K Thr389 phosphorylation than the IC group. However, the phosphorylation of other mTORC1-associated proteins (4E-BP1 and ribosomal protein S6) and the MPS response did not differ between the contraction modes. After the 13th bout of RE, mTORC1 activation and the MPS response were significantly blunted as compared with the 1st bout of RE. Changing from IC to EC did not improve these responses. In conclusion, changing the contraction mode to EC does not reinvigorate the blunted mTORC1 activation and MPS in response to RE during chronic RT.

## Introduction

Resistance exercise is known to stimulate muscle protein synthesis (MPS), and chronic resistance exercise training induces muscle hypertrophy (Ogasawara et al., 2016). Muscle hypertrophy is believed to occur as increased MPS enables new myofibrils to be added to pre-existing muscle fibers (Glass, 2003). Although the detailed molecular mechanisms of the resistance exercise-induced increase in MPS remain unclear, recent research revealed that the both rapamycin-sensitive and -insensitive mechanistic target of rapamycin complex (e.g., mTORC1 and/or mTORC2) plays a role in this event (Ogasawara et al., 2016; West et al., 2016; Ogasawara and Suginohara, 2018). Several studies have reported that acute resistance exercise induces mTORC1 activation, which is typically evaluated by measuring the phosphorylation of its downstream targets, such as p70S6K and 4E-BP1 (Burd et al., 2010a,b).

The time course of muscle hypertrophy by resistance training has been well studied. In general, muscle hypertrophy is greater during the early phase of resistance training (e.g., up to 2–3 months) than during the later phase (Woolstenhulme et al., 2006; Ogasawara et al., 2013b; Brook et al., 2015). Similarly, recent studies have reported that both mTORC1 activation and the MPS response are greater during the early phase of resistance training than during the later phase (Ogasawara et al., 2013a; Brook et al., 2015). Therefore, although the MPS response in the early phase of resistance training may contribute not only to muscle hypertrophy, but also to remodeling of muscle structure (Damas et al., 2016), greater stimulation of mTORC1 and MPS by acute resistance exercise should contribute to continuous muscle hypertrophy during the later phase of resistance training.

Essentially, muscle contraction during exercise is classified into three modes: eccentric (EC), concentric, and isometric (IC). A few previous studies have reported that EC has no or slightly positive effects on acute contraction-induced increases in MPS in untrained subjects (Phillips et al., 1997; Moore et al., 2005). However, some studies have reported that EC can induce greater mTORC1 activation (i.e., p70S6K phosphorylation) as compared with the other modes of contraction (Eliasson et al., 2006; Burry et al., 2007; Ato et al., 2016; Ashida et al., 2018). These observations indicate the possibility that EC reactivates mTORC1 signaling during chronic resistance training, resulting in greater increases in MPS. Therefore, we hypothesized that changing the contraction mode during resistance training could recover post-exercise mTORC1 activation and the MPS response. To test this hypothesis, we evaluated the influence of changing the contraction mode from IC to EC after chronic resistance training on post-exercise mTORC1 activation and MPS using a rat model of resistance exercise.

## Materials and Methods

### Animal Experimental Procedures

**FIGURE 1:**
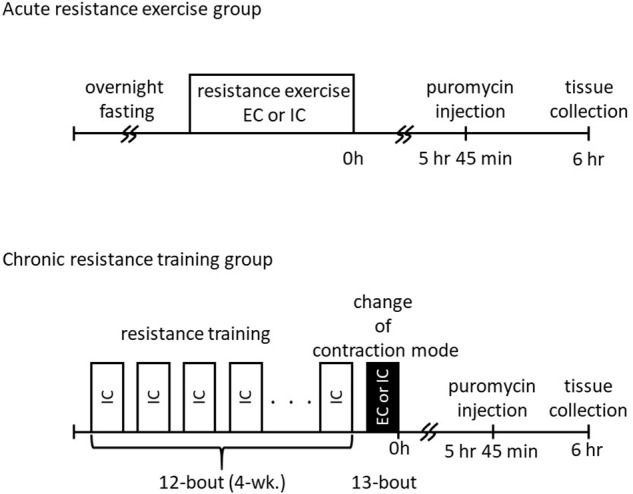
Schematic overview of study design.

The study protocol was approved by the Ethics Committee for Animal Experiments at Nippon Sport Science University, Japan. Twenty male Sprague–Dawley rats, aged 10 weeks (350–390 g body weight), were purchased from CREA Japan (Tokyo, Japan). The animals were housed for 1 week in an environment maintained at 22–24°C with a 12 h–12 h light–dark cycle and received food and water *ad libitum*. The rats were randomly classified into an acute resistance exercise group (one bout of resistance exercise; *n* = 10) and a chronic resistance training group (13 bouts of resistance exercise; *n* = 10). Additionally, each of these groups was subdivided into an IC group and an EC group (*n* = 5 per group). The acute resistance exercise group performed a single bout of unilateral exercise. The chronic resistance training group performed 12 bouts of unilateral training using IC. For the 13th bout of exercise, the IC subgroup performed a further bout of exercise using IC and the EC subgroup performed a further bout using EC (experimental scheme was shown in Figure 1). The rats in all groups were sacrificed by blood removal from the aorta, and gastrocnemius muscle was removed at 6 h after the final bout of exercise. The tissue samples were quickly frozen in liquid nitrogen and stored at −80°C until analysis.

### Resistance Exercise Training Protocols

#### Acute Resistance Exercise

After an overnight fast, the lower legs of each rat were shaved under inhaled isoflurane anesthesia. The rats were then positioned with their right foot on a footplate in the prone posture. The triceps surae muscle was stimulated percutaneously with disposable electrodes (Vitrode V, Nihon Kohden, Tokyo, Japan), which were cut to 10 mm × 5 mm size and connected to an electrical stimulator and an isolator. The right gastrocnemius muscle was exercised with five sets of muscle contraction separated by rest intervals of 3 min. Each set comprised 3 s stimulation × 10 contractions, with a 7 s interval between contractions. The non-exercised left gastrocnemius muscle served as the internal control. The voltage (∼30 V) and stimulation frequency (100 Hz) were adjusted to produce maximal isometric tension. The contraction mode was switched by changing the foot-tibia angle during contraction (60°–105°; the joint angular velocity was set at 15°/s in the EC group).

#### Chronic Resistance Training

Chronic training was performed as previously described (Ogasawara et al., 2016). In brief, acute resistance exercise using IC was performed every other day (e.g., either Monday, Wednesday, and Friday or Tuesday, Thursday, and Saturday) for 4 weeks (12 bouts in total). After the overnight fast, a 13th bout of exercise was performed using either IC or EC.

### Western Blotting

Western blot analysis was performed as reported previously (Ogasawara et al., 2016). Briefly, frozen muscle samples were powdered using a bead crusher (μT-12, TAITEC, Saitama, Japan), and 20 mg of each powdered sample was homogenized in 10 volumes of homogenization buffer containing 20 mM Tris-HCl (pH 7.5), 1% Nonidet^TM^ P40, 1% sodium deoxycholate, 1 mM EDTA, 1 mM EGTA, 150 mM NaCl, and Halt^TM^ protease and phosphatase inhibitor cocktail (Thermo Fisher Scientific, Waltham, MA, United States). Homogenates were centrifuged at 10,000 × *g* for 10 min at 4°C and the supernatants were collected. The protein concentration of each sample was then determined using the Protein Assay Rapid kit (Wako Pure Chemical Industries, Osaka, Japan). The samples were diluted in Laemmli sample buffer and boiled at 95°C for 5 min. Using 5–20% SDS-polyacrylamide gels, both 20 and 50 μg of protein were separated by electrophoresis and subsequently transferred to polyvinylidene difluoride membranes. After transfer, the membranes were washed in Tris-buffered saline containing 0.1% Tween^®^20 and then blocked with the Bullet Blocking One for Western Blotting buffer (Nacalai Tesque, Kyoto, Japan) for 5 min at room temperature. After blocking, the membranes were washed and incubated overnight at 4°C with primary antibodies including p-p70S6K (Thr389, cat#9205; Ser421/Thr424, cat#9204, Cell Signaling Technology, Danvers, MA, United States), p-ribosomal protein S6 (Ser235/236, cat#2211; Ser240/244, cat#2215, Cell Signaling Technology), and total-4E-BP1 (cat#9452, Cell Signaling Technology), p-FAK (Tyr397, cat#3283), p-p38MAPK (Thr180/Tyr182 cat#9211, Cell Signaling Technology), embryonic myosin heavy chain (cat#cs-53091, Santa Cruz Biotechnology, Dallas, TX, United States). The membranes were then washed again in Tris-buffered saline containing 0.1% Tween^®^ 20 and incubated for 1 h at room temperature with the appropriate secondary antibodies. Chemiluminescent reagents (ImmunoStar^®^LD, Wako Pure Chemical Industries) were used to facilitate the detection of protein bands. Images were scanned using a chemiluminescence detector (C-DiGit^®^blot scanner, LI-COR Biosciences, Lincoln, NE, United States). Band intensities were quantified using Image Studio^TM^ Lite Ver. 5.2 (LI-COR Biosciences). After the chemiluminescence detection, membranes were stained with Coomassie Brilliant Blue (CBB) solution, and the intensity of each protein band was normalized to that of the stained blot.

### Muscle Protein Synthesis

The *in vivo* SUnSET technique was used for MPS measurements (Goodman et al., 2011). Briefly, 0.04 μmol puromycin/g body weight (Wako, Tokyo, Japan) diluted in 0.02 M PBS was injected intraperitoneally under anesthesia 15 min before harvest. Following homogenization as described above and centrifugation at 2,000 × *g* for 3 min at 4°C, the supernatant was collected and processed for Western blotting. A mouse monoclonal anti-puromycin antibody (cat#MABE343, Millipore, Billerica, MA, United States) was used to detect puromycin-labeled nascent polypeptides, and the sum of the intensities of the protein ladder bands on each Western blot was evaluated.

### Statistical Analyses

Two-way ANOVA (contraction mode × training) was used to evaluate changes in the phosphorylation and/or expression of proteins. *Post hoc* analyses were performed using *t*-tests, with Benjamini–Hochberg false discovery rate correction for multiple comparisons when appropriate. All values were expressed as means ± standard error. The level of significance was set at *p* < 0.05.

## Results

**FIGURE 2:**
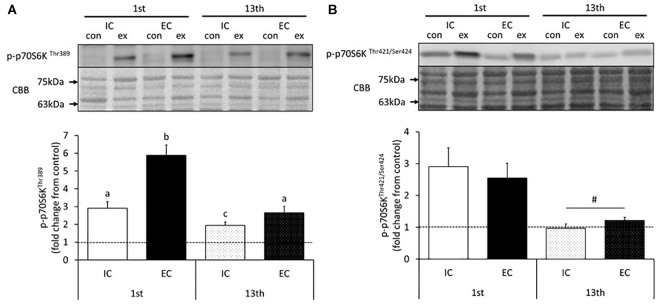
Phosphorylation of p70S6K on Thr389 **(A)** and Thr421/Ser424 **(B)** after the 1st and 13th bouts of resistance exercise. Values are means ± standard error (SE). Different letters indicate significant differences. ^#^Main effect of training (*p* < 0.05).

The changes in p70S6K phosphorylation in response to acute muscle contraction at the 1st and 13th bouts of exercise are shown in Figure 2 and Supplementary Figures S1, S2. The Thr389 residue of p70S6K became significantly phosphorylated in both the IC and EC groups at the 1st bout of exercise (Supplementary Figure S1, *p* < 0.05 vs. control). The level of phosphorylation was significantly higher in the EC group as compared with the IC group after the 1st bout (Figure 2A, IC: 291 ± 30%, EC: 588 ± 58%. *p* < 0.05 vs. IC). At the 13th bout of exercise, chronic resistance training significantly reduced Thr389 phosphorylation in response to acute muscle contraction in both the IC and EC groups as compared with their responses at the 1st bout (IC: 195 ± 19%, EC: 265 ± 36%). Additionally, the EC group did not exhibit a significantly different response from that of the IC group at the 1st bout. In contrast to the 1st bout of exercise, there was no significant difference in phosphorylation between the IC and EC groups at the 13th bout of exercise (Figure 2A).

The phosphorylation of the p70S6K Thr421/Ser424 residues significantly increased in both the IC and EC groups in response to the 1st bout of exercise (Supplementary Figure S2, *p* < 0.05 vs. control). The magnitude of phosphorylation did not differ between the IC and EC groups (IC: 290 ± 59%, EC: 255 ± 46%). At the 13th bout of exercise, no change in Thr421/Ser424 phosphorylation was detected in either group (Figure 2B and Supplementary Figure S2). Additionally, in both groups, Thr421/Ser424 phosphorylation in the exercised leg was significantly reduced as compared with that measured after the 1st bout of exercise (Figure 2B, *p* < 0.05).

**FIGURE 3:**
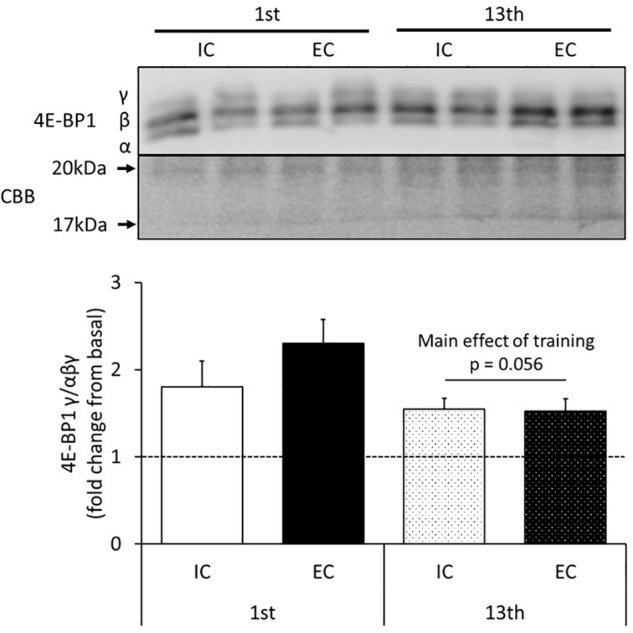
The expression of the γ isoform of 4E-BP1 after the 1st and 13th bouts of exercise. Values are means ± SE.

The changes in the activity of 4E-BP1 in response to acute muscle contraction at the 1st and 13th bouts of exercise are shown in Figure 3 and Supplementary Figure S3. The expression of the γ isoform of 4E-BP1 significantly increased in both the IC and EC groups after the 1st bout of exercise with no statistical difference between group (Supplementary Figure S3, IC: 180 ± 30%, EC: 230 ± 27%. *p* < 0.05 vs. control). After chronic resistance training, neither the IC nor EC group showed a difference in the expression of the γ isoform of 4E-BP1 as compared with the contralateral leg (Figure 3 and Supplementary Figure S3).

**FIGURE 4:**
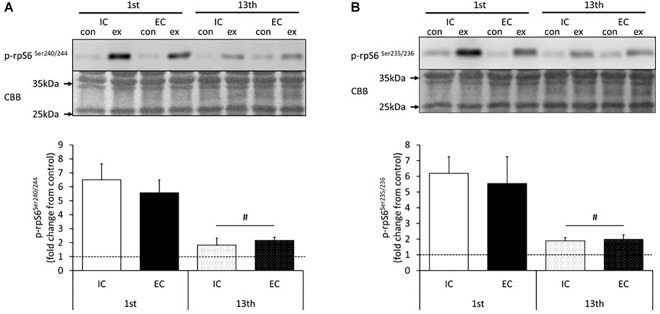
Phosphorylation of ribosomal protein S6 on Ser240/244 **(A)** and Ser235/Ser236 **(B)** after the 1st and 13th bouts of exercise. Values are means ± SE. ^#^Main effect of training (*p* < 0.05).

The results for the induction of ribosomal protein S6 phosphorylation by acute resistance exercise at the 1st and 13th bouts of exercise are shown in Figure 4 and Supplementary Figures S4, S5. At the 1st bout of exercise, the Ser240/244 residues of ribosomal protein S6 were significantly phosphorylated in both the IC and EC groups (Supplementary Figure S4, *p* < 0.05 vs. control). The level of phosphorylation was similar between the IC and EC groups (Figure 4A, IC: 907 ± 122%, EC: 711 ± 52%). At the 13th bout of exercise, both the IC and EC groups exhibited significantly increased phosphorylation on the Ser240/244 residues to the same degree (Figure 4A and Supplementary Figure S4, IC: 361 ± 54%, EC: 311 ± 41%. *p* < 0.05 vs. control). In both groups, the magnitude of phosphorylation was significantly lower as compared with that measured at the 1st bout of exercise (Figure 4A, *p* < 0.05).

In addition, the Ser235/236 residues of ribosomal protein S6 were significantly phosphorylated in both the IC and EC groups at the 1st bout of exercise to the same extent (Figure 4B and Supplementary Figure S5, IC: 619 ± 106%, EC: 553 ± 172%). At the 13th bout of exercise, Ser235/236 phosphorylation was significantly lower in both groups as compared with the 1st bout (Figure 4B and Supplementary Figure S5, IC: 189 ± 21%, EC: 199 ± 28%. *p* < 0.05). The level of phosphorylation did not differ among groups at the 13th bout of exercise.

**FIGURE 5:**
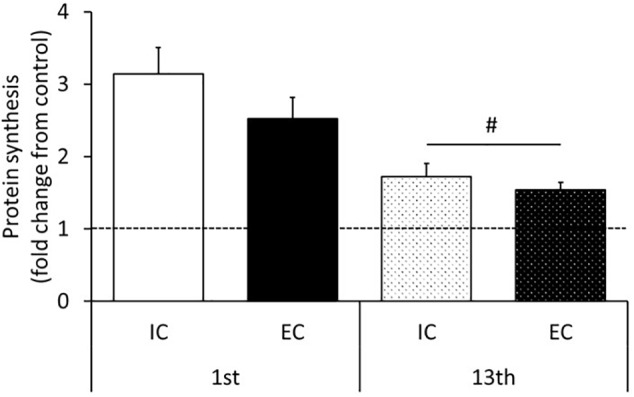
Muscle protein synthesis after the 1st and 13th bouts of exercise. Values are means ± SE. Values are means ± SE. ^#^Main effect of training (*p* < 0.05).

The rates of MPS after the 1st and 13th bouts of exercise are shown in Figure 5 and Supplementary Figure S6. MPS significantly increased after the 1st bout in both the IC and EC groups to a similar degree (Figure 5 and Supplementary Figure S6, IC: 314 ± 36%, EC: 252 ± 30%, *p* < 0.05 vs. control). At the 13th bout of exercise, MPS significantly increased in both the IC and EC groups to the same extent (Figure 5 and Supplementary Figure S6, IC: 172 ± 19%, EC: 154 ± 11%, *p* < 0.05 vs. control). Moreover, the exercise-induced the MPS response was significantly lower at the 13th bout as compared with the 1st bout of exercise in both the IC and EC groups (Figure 5, *p* < 0.05).

## Discussion

In this study, we investigated the influence of changing muscle contraction modes from IC to EC after chronic resistance training on the post-exercise responses of mTORC1 activation and MPS. We hypothesized that since there is a possibility that EC might stimulate mTORC1 and MPS to a greater extent than IC, EC might therefore reinvigorate blunted mTORC1 activation and MPS after chronic training. However, acute EC enhanced only p70S6K phosphorylation and this did not contribute to the MPS response. Moreover, changing the contraction mode to EC failed to reinvigorate blunted mTORC1 activation and MPS in response to acute exercise during a period of chronic training.

### Effect of EC on the MPS Response After Acute Resistance Exercise

Previous studies have reported that EC potently induces higher mTORC1 activation and MPS than other modes of contraction in rodents and humans (Moore et al., 2005; Eliasson et al., 2006; Ato et al., 2016). Similarly, we observed in the present study that acute exercise-induced p70S6K Thr389 phosphorylation was higher in the EC group than in the IC group after 1st bout of exercise. Thus, it is apparent that acute EC potently augments p70S6K Thr389 phosphorylation. p70S6K on Thr421/Ser424 is involved in the initiation of kinase activation through the release of kinase autoinhibition by phosphorylation (Martin et al., 2014). In this study, the phosphorylation status of p70S6K Thr421/Ser424 did not statistically differ between the IC and EC groups after the 1st bout of exercise. Additionally, we investigated the downstream substrates of p70S6K and found that the phosphorylation of ribosomal protein S6 Ser240/244 and Ser235/236 increased after acute exercise to a similar extent in both the IC and EC groups after 1st bout of exercise. Therefore, the present results suggested that although EC more strongly induces the phosphorylation of p70S6K Thr389 than IC does, the activity of p70S6K might not robustly differ between these contraction modes.

Secondly, the mTORC1 target substrate, 4E-BP1, is usually bound to eIF4E and limits translation at ribosomes (Gautsch et al., 1998). Acute resistance exercise inactivates 4E-BP1, causing it to dissociate from eIF4E, resulting in increased MPS (Kubica et al., 2005; Liu et al., 2013; Ogasawara et al., 2016). In this study, the expression of the 4E-BP1γ isoform, which indicates 4E-BP1 dissociation from eIF4E, was increased after acute resistance exercise in agreement with previous observations (Ogasawara et al., 2016, 2017). However, unlike p70S6K Thr389 phosphorylation, the expression level of the 4E-BP1 γ isoform did not differ between the IC and EC groups after exercise. In a previous study by another group, it was observed that muscle contraction induced p70S6K phosphorylation and 4E-BP1 inactivation to different degrees (Liu et al., 2013). In addition, we recently demonstrated that p70S6K Thr389 phosphorylation is predominantly regulated by rapamycin-sensitive mTORC, but 4E-BP1 is controlled by both rapamycin-sensitive mTORC1 and rapamycin-insensitive mTORC in skeletal muscle (Ogasawara and Suginohara, 2018; You et al., 2018). Therefore, our results indicate that EC might specifically stimulate rapamycin sensitive mTORC1 via unknown mechanisms after 1st bout of exercise in this study.

In the current study, the rate of MPS did not differ between the IC and EC groups after 1st bout of exercise, as is also the case in previous studies (Phillips et al., 1997; Cuthbertson et al., 2006; Franchi et al., 2018). Classically, the level of p70S6K Thr389 phosphorylation is known to correspond with MPS after acute RE. However, recent studies have observed that the MPS response after RE does not necessarily reflect the level of p70S6K phosphorylation, suggesting that rapamycin-sensitive mTORC plays a minor role in the regulation of MPS after muscle contraction (Ogasawara and Suginohara, 2018; You et al., 2018). Therefore, although EC simulates rapamycin-sensitive mTORC1 to a greater extent than IC, it does not induce greater increases in MPS.

On the other hand, we previously reported that the MPS response to IC gradually increased with set number (i.e., force-time integral) and then plateaued at five sets in our experimental model (Ogasawara et al., 2017). Therefore, the MPS response to muscle contraction may have reached plateau.

### Effect of Chronic Resistance Training on the MPS Response After Acute Exercise

Muscle hypertrophy in response to resistance training is robust during the early phase of training and subsequently wanes with continuous training (Ogasawara et al., 2013a; Brook et al., 2015). It has previously been reported that the induction of mTORC1 activation and MPS in response to acute exercise is blunted by chronic resistance training, and this phenomenon is associated with a stagnation of muscle hypertrophy during chronic resistance training (Ogasawara et al., 2013a; Brook et al., 2015). Concordantly, we observed in the current study that the activation of downstream targets of mTORC1 in response to acute exercise was significantly reduced after 13 bouts of exercise as compared with the 1st bout of exercise. Taken together, previous studies and our current results suggest that reduced activation of mTORC1-regulated molecules is involved in the blunted the MPS response during continuous resistance training, and this may lead to a plateau of muscle hypertrophy.

Previous studies have suggested that the MPS response in the early phase of resistance training is accompanied by muscle damage and/or remodeling (Damas et al., 2016). However, in this study, we could not detect any change in the molecules that associate with tissue inflammation or muscle structure remodeling (p38MAPK, FAK) to acute exercise after both 1st and 13th bouts of exercise (Supplementary Figures S7, S8). In addition, although we measured embryonic myosin heavy chain as a muscle damage marker, no obvious band could be detected in the present study (data not shown). Thus, our results indicate that these responses may not necessarily be related to higher mTORC1 activation and MPS during acute exercise in the early phase of training. However, it should be noted that although the sampling point in this study was 6 h post exercise, previous studies have shown that acute resistance exercise increases the phosphorylation of p38MAPK and FAK only during the early phase of recovery (<2 h post exercise) (Wilkinson et al., 2008; Moller et al., 2013; Figueiredo et al., 2016). Previous studies have also observed that EC greatly increases p38MAPK phosphorylation (Franchi et al., 2014). In addition, FAK phosphorylation is also strongly stimulated by EC (Gehlert et al., 2014). Therefore, we could not exclude the possibility that these molecules contribute to mTORC1 activation and the MPS response during the early phase of recovery after resistance exercise. Future study is needed to investigate whether the inhibition of those molecules during and after resistance exercise affects mTOR activation and MPS.

### Effect of Changing Contraction Mode on the MPS Response to Acute Resistance Exercise During Successive Resistance Training

The primary purpose of this study was to evaluate whether changing the contraction mode from IC to EC could rescue the acute exercise-induced the MPS response during chronic resistance training. Considering previous reports that EC has potential to stimulate mTORC1 and MPS (Moore et al., 2005; Eliasson et al., 2006; Ato et al., 2016; Ashida et al., 2018), we hypothesized that changing the contraction mode to EC during chronic resistance training may reinvigorate mTORC1 activation and the MPS response to exercise. In the current study, however, changing contraction mode did not re-invigorate mTORC1 activation and MPS in response to acute exercise during chronic resistance training. Interestingly, even though EC produced significantly greater p70S6K Thr389 phosphorylation than IC after 1st bout of exercise, this difference disappeared after chronic resistance training. Furthermore, mTORC1 activation after EC was blunted after 13 bouts compared with after just one bout, even though the first 12 bouts of chronic resistance training were performed using IC. Recent studies also observed that in humans, resistance exercise-induced the MPS response is blunted by chronic resistance training with no relation to distinct contraction modes (Franchi et al., 2018). Therefore, these results indicate that chronic resistance training reduced the responsivity of mTORC1 activation to exercise independently of the contraction mode and that changing the contraction mode from IC to EC is not suitable for improving mTORC1 activation and the MPS response to acute resistance exercise during continuous resistance training.

Previous studies and the results herein show that chronic high force contractile activity such as resistance training, diminishes mTORC1 activation and MPS in response to acute exercise (Ogasawara et al., 2013a; Brook et al., 2015; Damas et al., 2015). Nevertheless, the mechanisms underlying these phenomena remain unclear. In contrast, a reduction in contractile activity (detraining or unloading) sensitizes mTORC1 activation and MPS in response to acute resistance exercise or intense muscle contraction (Ogasawara et al., 2013a; You et al., 2015). Hence, the constitutive muscle contractile status may modulate the responsivity of mTORC1 and MPS to acute exercise. Clarification of the mechanisms connecting chronic contractile activity and the sensitivity of the MPS response to acute muscle contraction should provide an efficient way to overcome the stagnation of muscle hypertrophy during chronic resistance training.

## Ethics Statement

The study protocol was approved by the Ethics Committee for Animal Experiments at Nippon Sport Science University, Japan.

## Author Contributions

KN and RO contributed to the conception and design of the experiments. SA, DT, YI, TS, YM, and RO collected, analyzed, and interpreted the data. SA, KN, and RO drafted the article and revised it critically for important intellectual content. All authors approved the final version of the manuscript and qualify for authorship.

## Conflict of Interest Statement

The authors declare that the research was conducted in the absence of any commercial or financial relationships that could be construed as a potential conflict of interest.
