# Supplementary material for: The Effect of Changing the Contraction Mode During Resistance Training on mTORC1 Signaling and Muscle Protein Synthesis
Source: Front Physiol. 2019 Apr 18;10:406. doi: 10.3389/fphys.2019.00406 (PMC6482468; doi:10.3389/fphys.2019.00406)
Supplement: Supplementary file 1 [file Table_1.DOCX]

Supplementary Material


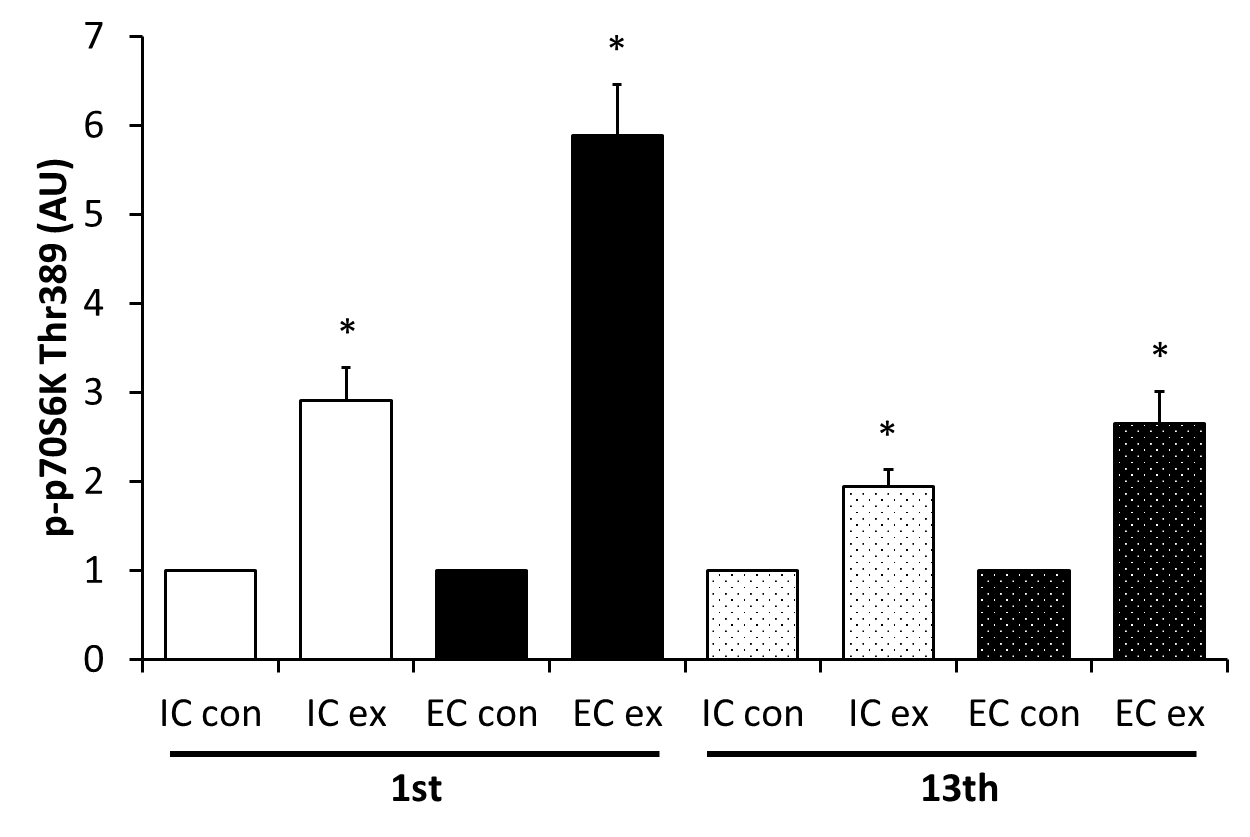


Supplementary Figure 1. Phosphorylation of p70S6K Thr389 after the 1^st^ and 13^th^ bouts of resistance exercise. Values are means ± standard error (SE). *: p < 0.05 vs. control leg.


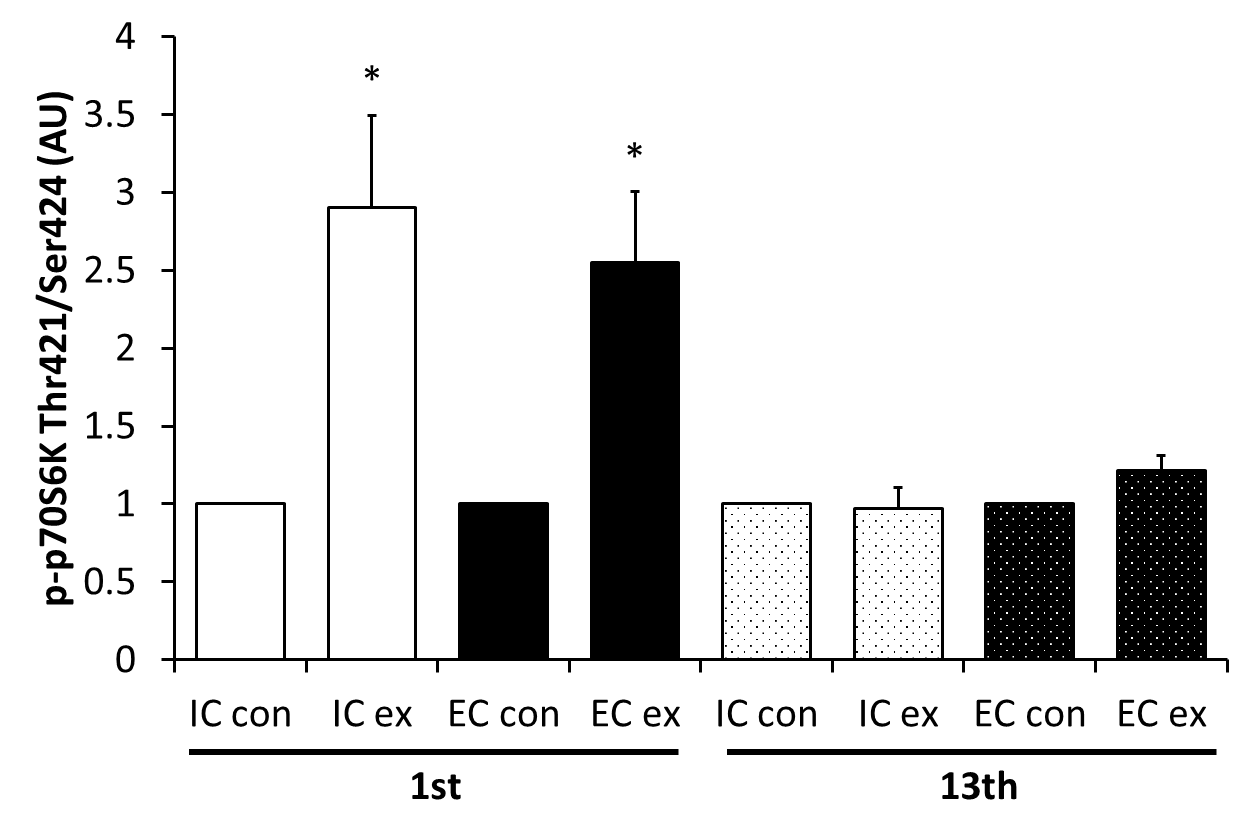


Supplementary Figure 2. Phosphorylation of p70S6K Thr421/Ser424 after the 1st and 13th bouts of resistance exercise. Values are means ± standard error (SE). *: p < 0.05 vs. control leg.


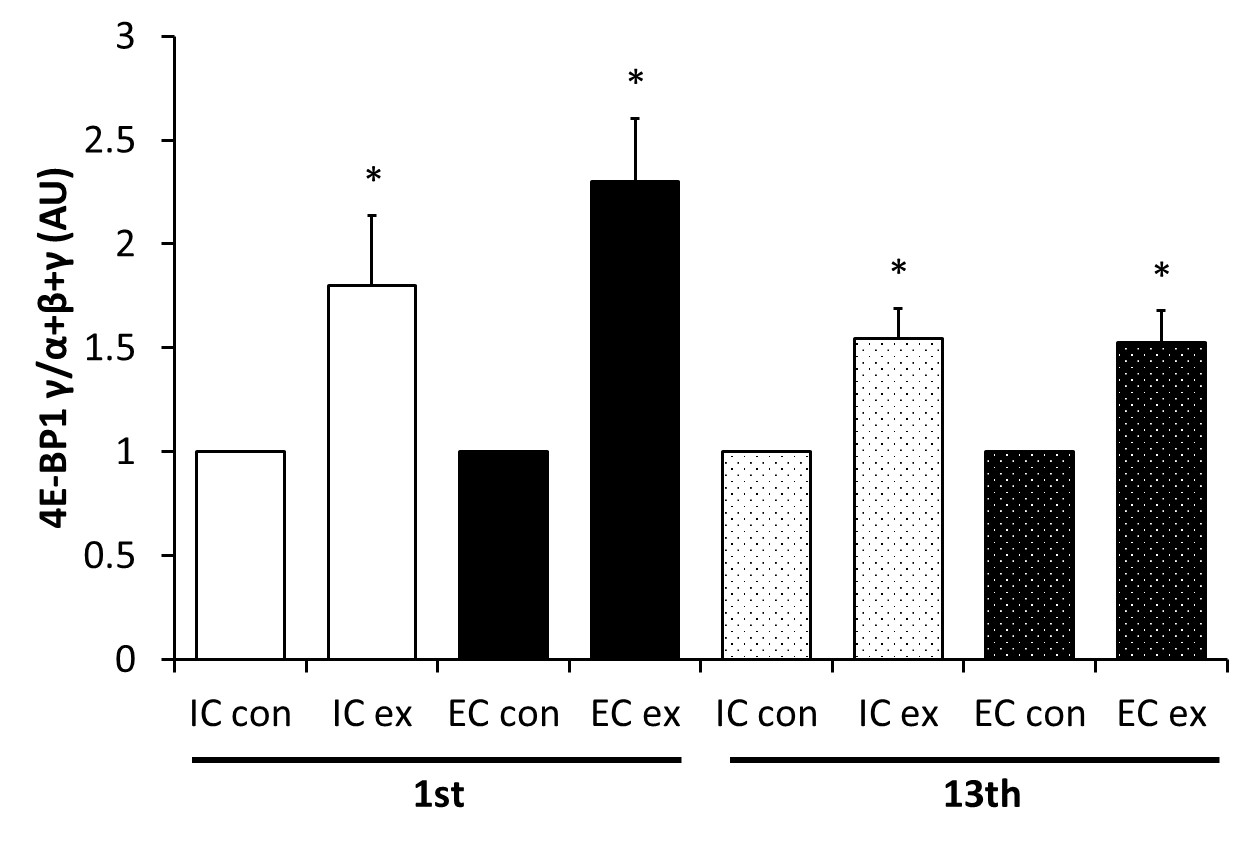


Supplementary Figure 3. Change in γ isoform of 4E-BP1 expression after the 1st and 13th bouts of resistance exercise. Values are means ± standard error (SE). *: p < 0.05 vs. control leg.


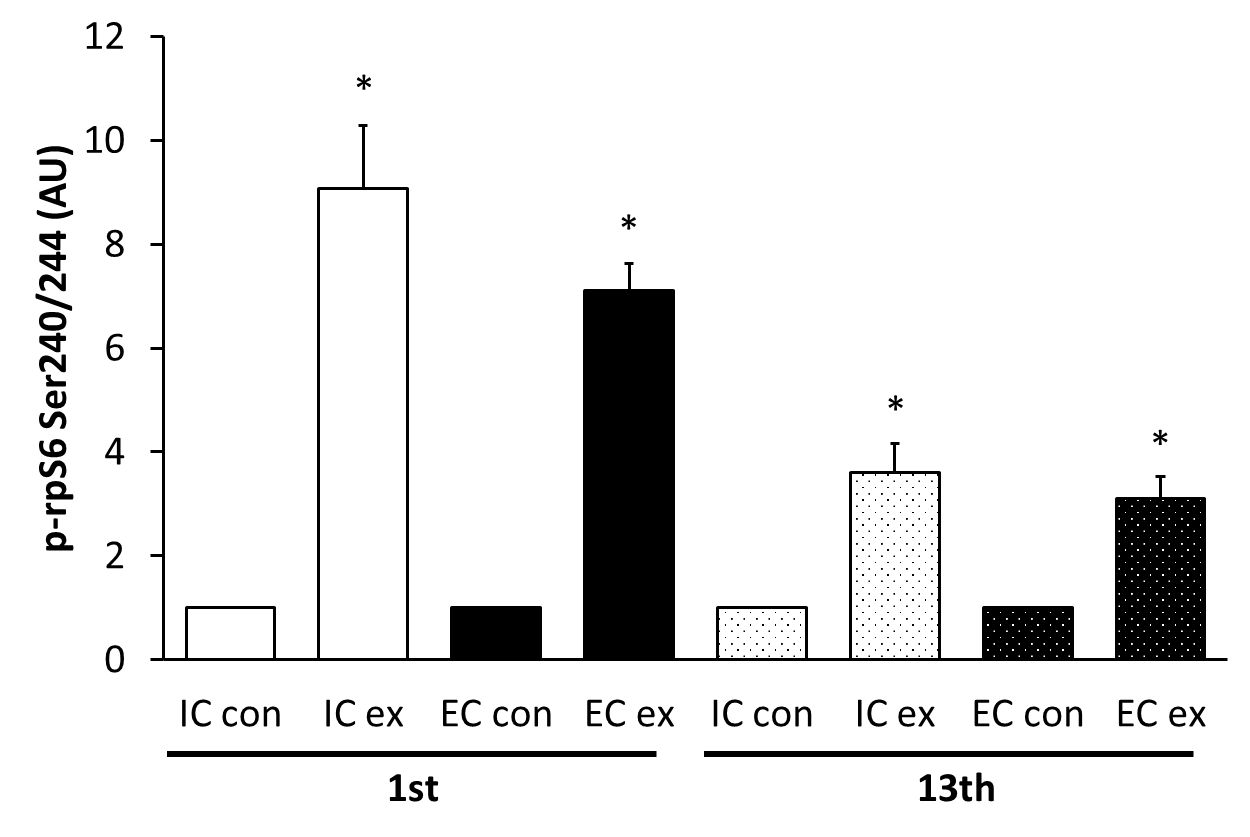


Supplementary Figure 4. Phosphorylation of ribosomal protein S6 Ser240/244 after the 1^st^ and 13^th^ bouts of resistance exercise. Values are means ± standard error (SE). *: p < 0.05 vs. control leg.


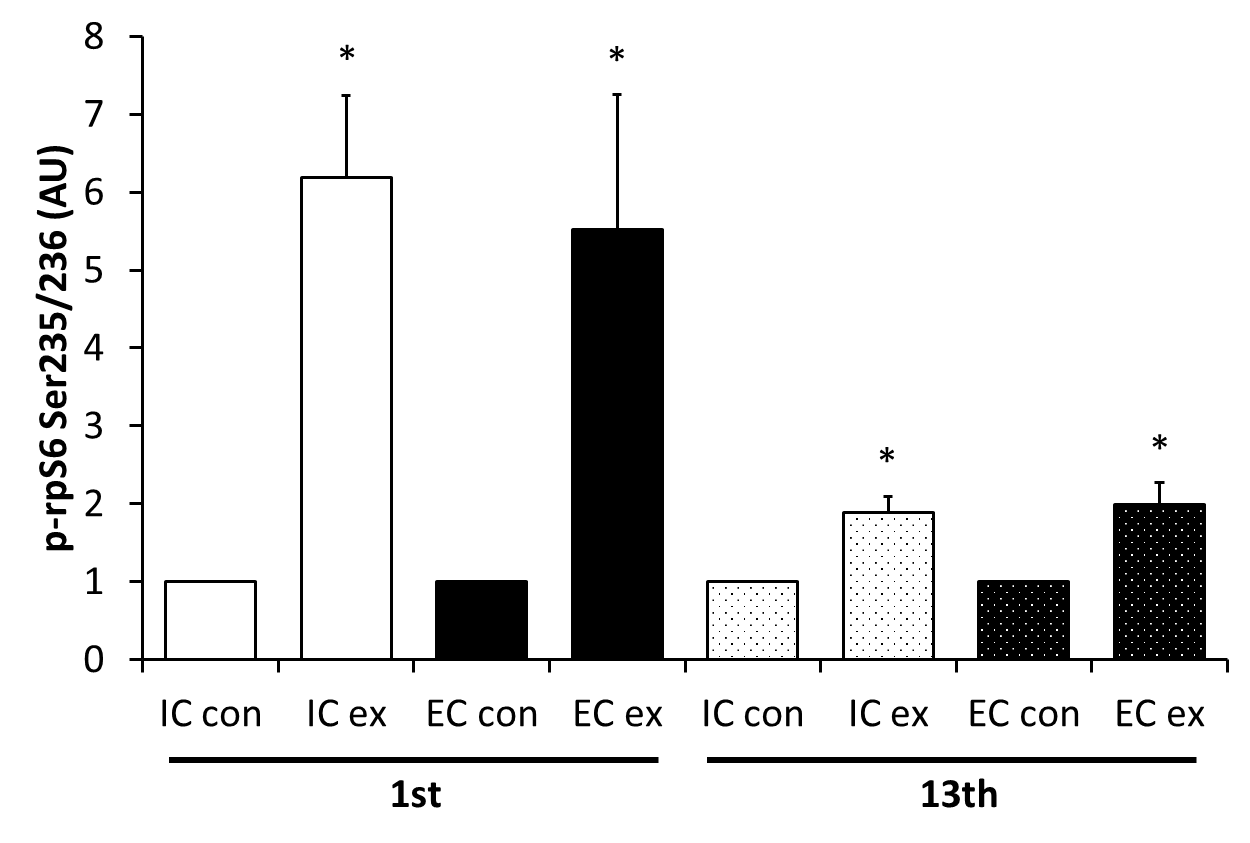


**Supplementary Figure 5.** Phosphorylation of ribosomal protein S6 Ser235/236 after the 1st and 13th bouts of resistance exercise. Values are means ± standard error (SE). *: p < 0.05 vs. control leg.


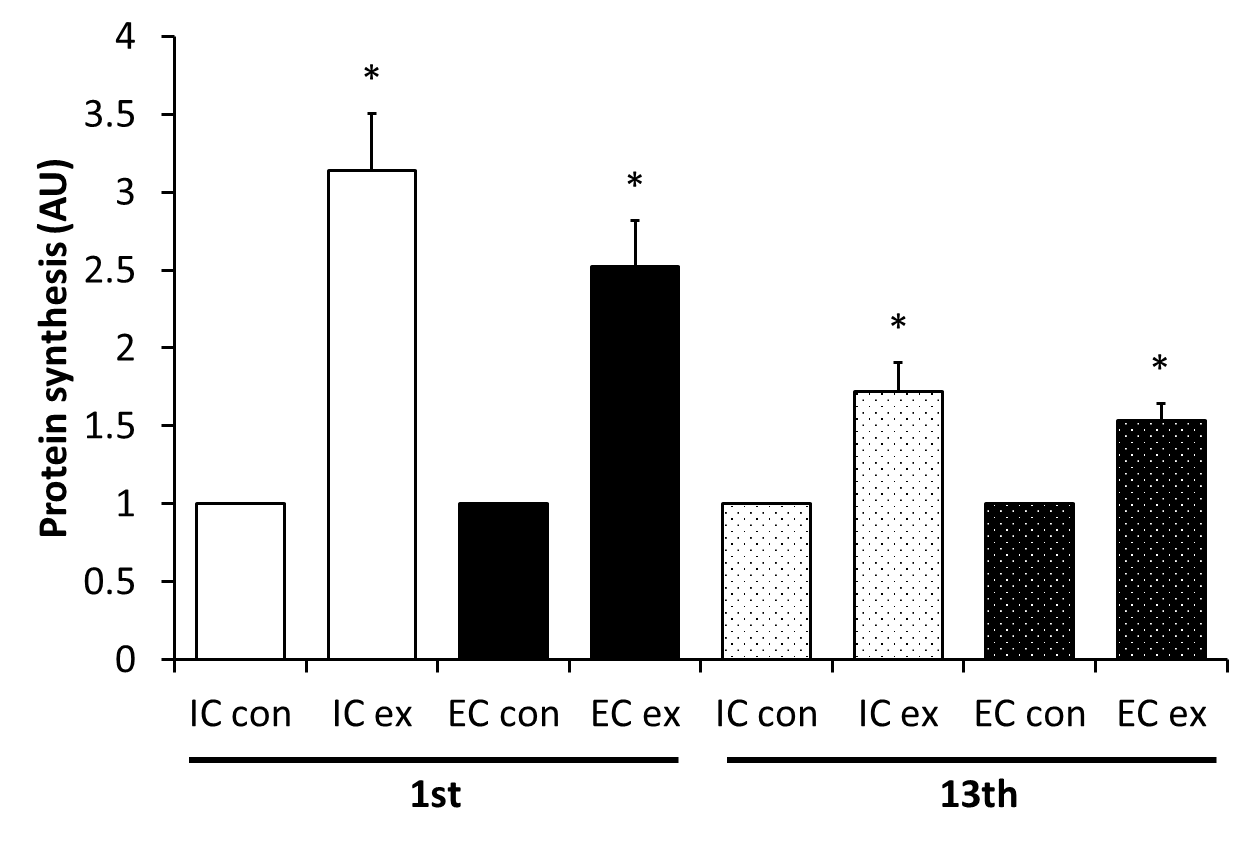


**Supplementary Figure 6.** Change in protein synthesis after the 1st and 13th bouts of resistance exercise. Values are means ± standard error (SE). *: p < 0.05 vs. control leg.


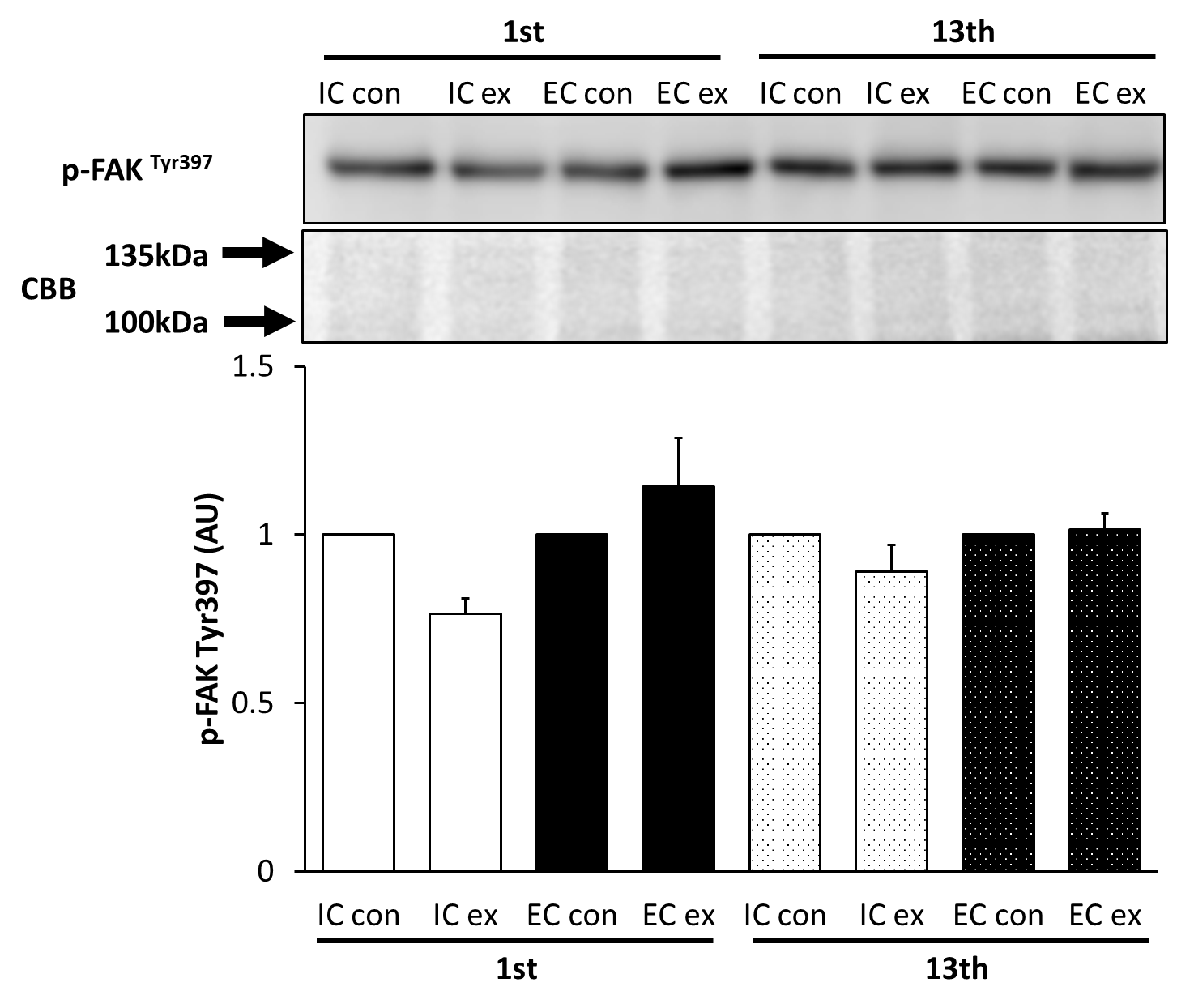


**Supplementary Figure 7.** Phosphorylation of FAK Tyr397 after the 1^st^ and 13^th^ bouts of resistance exercise. Values are means ± standard error (SE).


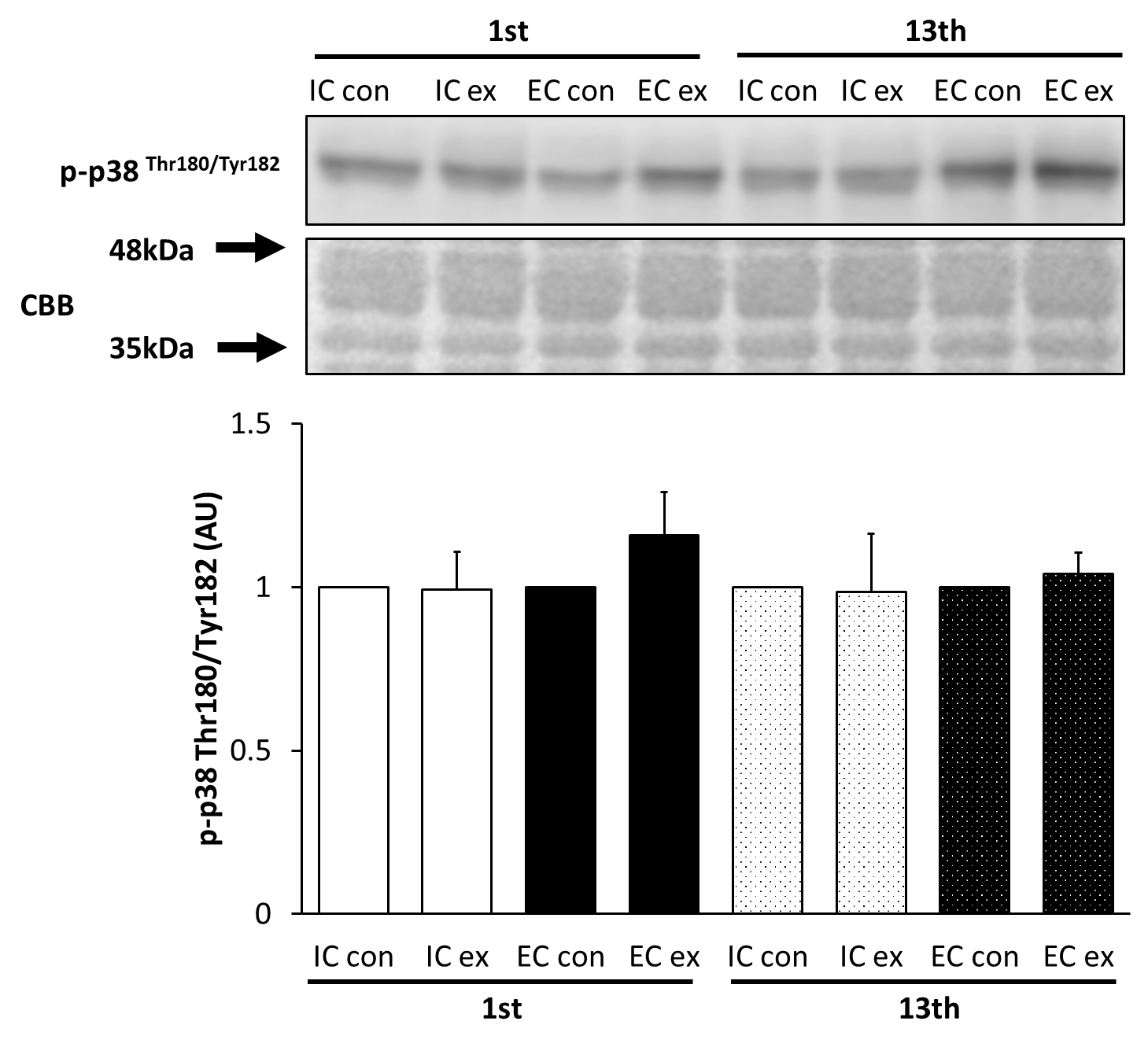


**Supplementary Figure 8.** Phosphorylation of p38MAPK Thr180/Tyr182 after the 1^st^ and 13^th^ bouts of resistance exercise. Values are means ± standard error (SE).
